# Experimental Analysis of the Mechanical Properties of Carbon Foams Under Quasi-Static Compressive Loads

**DOI:** 10.3390/ma17225605

**Published:** 2024-11-16

**Authors:** Krzysztof Wacławiak, Jerzy Myalski, Debela N. Gurmu, Goftila G. Sirata

**Affiliations:** Faculty of Materials Engineering, Department of Materials Technologies, Silesian University of Technology, ul. Krasińskiego 8, 40-019 Katowice, Poland; krzysztof.waclawiak@polsl.pl (K.W.); jerzy.myalski@polsl.pl (J.M.); goftila.gudeta.sirata@polsl.pl (G.G.S.)

**Keywords:** cellular materials, carbon foam, carbonization, failure mechanism

## Abstract

This article sought to determine the response of a carbon foam material derived from polyurethane foam when subjected to a quasi-static compression load. The effects of the foam pore densities and additives (solvents) on the compression strength, compressive modulus, and surface morphology of the carbon foam were investigated. In this study, three different carbon foam pore densities (20, 40, and 60 ppi) and three solvents for the phenol–formaldehyde resins that coated the polymer foam (acetone, ethanol, and methanol) were used. Carbon foams were derived from polyurethane foams by carbonization. Quasi-static compression testing was carried out using a universal testing machine. The compressive strength, compressive modulus, and relative density of these different carbon foams were computed and compared. Two-way ANOVA analyses were performed to compare the significance of solvents and pore density. These results showed that pore density and solvents significantly affected the compressive strength, compressive modulus, and surface morphology of the fabricated polyurethane-derived carbon foam. Finally, the maximum compressive strength and maximum compressive modulus were observed in carbon foam (60 ppi) with 40% methanol as the solvent. Conversely, a minimum compressive strength was observed for a 20 ppi carbon foam with a 20% acetone solvent, and a minimum compressive modulus was observed for a 20 ppi foam with 40% methanol. Lastly, the chemical composition of the polyurethane foams was investigated, and these results indicated that the polyurethane-derived carbon foam had 96% carbon atoms after carbonization.

## 1. Introduction

Carbon foams are 3D structural and sponge-like carbon materials [1,2,3] and have attracted increasing attention from academic communities and industry because of their excellent performance characteristics, such as low density, high adsorption, and large surface area [4,5], as well as good electromagnetic and acoustic properties [6]. Carbon foams are used in many areas, such as the electronics industry, automotive industry, construction industry [7], aerospace, rocket nozzles, motors, bone surgery, prosthetics, tooth implant materials, energy storage [8], fuel cells, padding materials, heat exchangers [9], high-temperature thermal insulation, battery electrodes, catalyst support, radar absorption, filters, and sensors [10,11,12]. Carbon foam is easily machined and provides a large surface area for bonding [4].

The properties of carbon foams primarily depend on fundamental parameters such as density, internal structure (mainly pore size and number of pores), and pore wall thickness [7,13]. On the other hand, factors such as pore size, pore density, and the foam geometry structure play a critical role in improving the mechanical properties of carbon foams [14]. Currently, all materials can be foamed, including metal [15,16], ceramics [17,18] and polymers [19]. Carbon foams produced from polymer precursors include resins, tannins, polyurethane, urethane [20,21,22], pitches [23,24,25] (coal tar pitch, petroleum asphalt, and naphthalene-based pitch), and biomass (flour, sucrose, bread, coconut shell, and wood) substances [26,27].

Carbon foam properties depend on several different factors. For example, Yan et al. [28] reported that the mechanical properties of carbon foam materials depend on the initial precursor compositions, foam densities, and the microstructural arrangement in the foams. Furthermore, Calvo et al. [29] stated that foaming pressure and temperature also affect the overall performance of a carbon foam by affecting the pore size and pore volume. Additionally, Baran et al. [30] report that the pore size and density of carbon foams also affect the compressive strength synthesized using an asphaltene pitch as a carbon precursor. In addition to these parameters, relative density also directly affects the compressive strength of coal-derived carbon foams as presented by Chen et al. [25]. Yao et al. [31] studied the effect of bulk density on the compressive strength and modulus of carbon foams manufactured from a sucrose/polyacrylamide hydrogel using gel casting and physical foaming. Those results showed that the compressive strength increased by 54.5% when the bulk density of the carbon foams increased from 530 to 590 kg/m^3^, and the compressive modulus also increased with increasing density. On the other hand, the particle size of the precursor, the heating rate during carbonization, and the foaming rate also affected the compressive strength of the carbon foam as reported by Banerjee et al. [32]. Fine particles and three hours of foaming yielded higher compressive strengths. Also, a heating rate of 3 K/min resulted in a higher bulk density and compressive strength than 5 K/min. This indicates that a slower heating rate reduces the possibility of crack formation and improves the foam compressive strength. The chemical composition, fluidity, and foaming pressure of the precursor also affect the structure and mechanical properties of carbon foams [33,34].

Another parameter that affects the mechanical properties (mainly the compressive properties) of carbon foams involves additives. Today, different additives are used to improve the properties of carbon foam. For example, Calvo et al. [23] investigated the effect of liptinite and vitrinite additives on the pore size of a carbon foam prepared from bituminous coal. Those results indicated the average pore size of the carbon foam increased with an increasing volume fraction of the liptinite but decreased with an increased volume fraction of the vitrinite in the precursor. Dang et al. [35] studied the effect of the mass fraction of phenol resin additives on the compressive strength of coal tar-derived carbon foam. The result showed a significant higher compressive strength compared to pure carbon foam without additives. In a similar manner, Wang et al. [36] used montmorillonite clay as an additive in a carbon foam derived from coal tar and studied the effect of the clay weight fraction on the carbon foam compressive properties. This resulted in an increase in the compressive strength of the carbon foam with an increase in the weight fraction of clay, and the thermal conductivity significantly decreased when the weight fraction of clay increased. The authors reported a maximum compressive strength when the clay content was 10% and a minimum compressive strength when the clay content 2%. In addition, Li et al. [37] studied the effect of boric acid additives on the microstructure and compressive strength of mesophase carbon foam. Those results indicated that the compressive strength initially increased with added boric acid, then decreased after the weight fraction was >5%, and minimized when the boric acid was 10%. In addition, boric acid as an additive affected the cell morphology in terms of cell wall thickness, pore section diameter, and foam density. On the other hand, Khan et al. [38] used multiwall nanotubes (MWNTs) and diamond nanoparticles (DNPs) as additives in mesophase coal tar pitch-derived carbon foams to investigate their effects on the compressive strength of mesophase coal tar pitch-derived carbon foams. Those results demonstrated that these additives much improved the compressive strength of the carbon foams. In a similar manner, Liu et al. [39] also studied the effect of multi-walled carbon nanotube (MWCNT) additives on the mechanical strengths of pitched-derived carbon foams. Those results showed that the ultimate compressive strength and the compressive Young’s modulus increased as the amount of MWCNTs was increased.

Nowadays, metals are seen as promising materials and are used as additives in carbon foams to enhance their properties. For example, Li et al. [40] studied the effect of titanium (Ti) additive on the microstructure and performance of carbon foams derived from mesophase pitch. The authors report that Ti promoted more perfect and larger crystallites and enhanced the conductive and mechanical properties of carbon foams. On the other hand, carbon nanotubes and montmorillonite (MMT) were used as additives, and the effect of these additives on the mechanical properties of carbon foam was reported by Wang et al. [41]. Their results indicated that the bulk density and compressive strength of the composite carbon foams increased with MMT amounts up to 5 wt.% and then decreased as the MMT content increased above 5 wt.%. Carbon foam composites containing 0.2 wt.% CNT and 5 wt.% MMT had the highest compressive strengths and bulk densities. Aluminium powder was also used as an additive and the weight fraction effect on the mechanical properties was studied by Farhan et al. [42]. Those results indicated that both the ultimate compressive strength and the bulk density of composite carbon foam increased as the aluminium powder content increased from 0 to 8 wt.%. The maximum ultimate compressive strength and bulk density were observed at 8 wt.% aluminium powder. The ultimate compressive strength and bulk density of the composite foam increased by 106% and 20% as compared to the pure carbon foam.

In this study, the effect of carbon foam pore density and additives to the phenol–formaldehyde resin (ethanol, methanol, and acetone) on the compressive strength and compressive modulus of carbon foams made from polyurethane foam was experimentally analyzed under a quasi-static uniaxial compressive load. A uniaxial compression test was performed with a 2.5 kN universal testing machine at room temperature at a loading rate of 5 mm/min. The load–displacement data during compression were obtained, and the nominal stress and nominal strain were calculated from these data. The strain was calculated as the ratio of displacement of the crosshead to the initial length of carbon foam specimens, while the stress was calculated as the ratio of the quasi-static load to the initial cross-sectional area of the carbon foam in all tests. In general, the stress–strain response was calculated from the applied load, the cross-sectional area, and the crosshead displacement. Both the compressive strength and the compressive modulus were determined from the stress–strain curves.

## 2. Materials and Methods

In this paper, carbon foams were made using the template method from a polyurethane polymer foam. The polyurethane polymer material was selected as a precursor for the carbon foam due to its advantages of low cost, easy processability, myriad design options, and that polyurethane foam (PUF) is a widely used material in many industries and daily life [43,44]. In addition, three solvents (acetone, methanol, and ethanol) were used for the phenol–formaldehyde resin to coat the polyurethane carbon foam. The compounds most used to dilute phenol–formaldehyde resins from a Polish manufacturer CHEMIPUR Lublin were used as solvents, which include polar compounds containing both alcohol-OH and the aliphatic ketone groups CH_2_ and C_2_H_3_ also found in unmodified phenol–formaldehyde resins. They were characterized primarily by a low boiling point below the cross-linking temperature of phenolic resins. This allowed evaporation at the precursor preparation stage before carbonization, i.e., resitol resin, in which evaporation of these compounds began before initial curing at 70 °C. The boiling point temperatures and their densities were as follows: for acetone 56 °C and 0.78 g/cm^3^; for methanol 64.7 °C and 0.792 g/cm^3^; and for ethanol 78 °C and 0.789 g/cm^3^. The choice of these types of solvents was determined by both their low environmental and health harmfulness.

### 2.1. Sample Preparation Procedure

**Figure 1:**
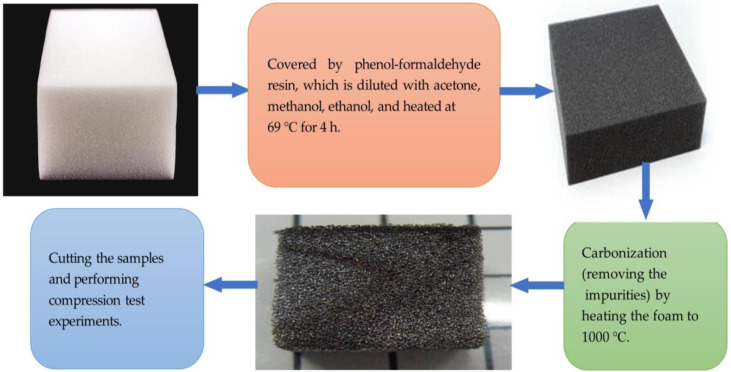
Sample preparation method.

Generally, carbon foam preparation processes can be classified as blowing, carbonization, template synthesis carbonization, compression of exfoliated graphite, assembly of graphene nanosheets, and others [1]. In this paper, direct carbonization was used for the preparation of carbon foam as shown in Figure 1. This is because carbonization is essential to obtain carbon foams from polymer precursors and results in higher applicability and efficiency than other methods. No blowing agent is needed for this method [5].

**Figure 2:**
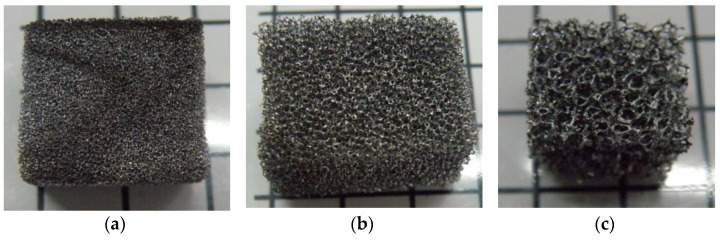
Carbon foam specimens of different density: (**a**) 60, (**b**) 40, and (**c**) 20 ppi.

In this article, 27 rectangular block specimens with length (l), width (w), and height (h) were obtained from large samples of carbon foam of different pore cell densities (20, 40, and 60 ppi) and manufactured with different solvents (acetone, methanol, and ethanol). Nine specimens were cut from high-pore-density carbon foam, expressed as 60 ppi (porous per inch) (Figure 2a), another nine from medium-pore-density (Figure 2b) carbon foam (40 ppi), and the other nine were from low-pore-density (Figure 2c) carbon foam (20 ppi). Each pore density had three specimens manufactured with 20% ethanol, three with 40% methanol, and three with 20% acetone as the solvent. This set of specimens was used to determine their compressive strength and compressive modulus. The size of the specimens was measured with vainer callipers. Another set of 9 specimens was used to determine the relative density and eventually the C_2_ constant in an equation describing the linear elastic properties of this cellular solid material. Their weight was measured with a digital weight balancing machine. After that, the volume and apparent density of the carbon foam were calculated, and the results are presented in Table 1.

### 2.2. Experimental Design

**Figure 3:**
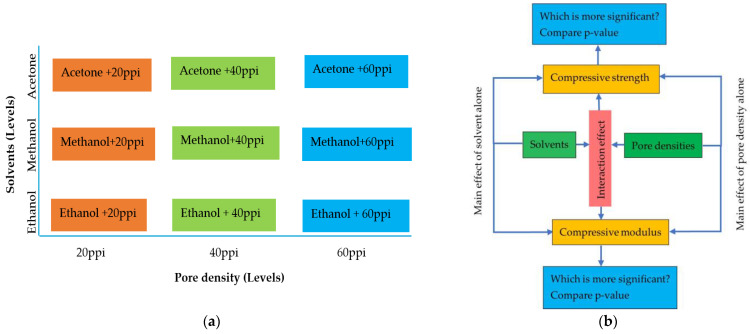
Experimental factorial design (**a**) and experimental design methodology (**b**).

In this article, two factorial designs (pore densities and solvents) were used to analyze the effect of pore density and solvents on both the compressive strength and compressive modulus of the carbon foams. Both the pore densities and solvents had three levels each. The levels of pore density were 20, 40, and 60 ppi, and solvent levels were 20% ethanol, 40% methanol, and 20% acetone. All possible combinations and experimental methodology were shown in Figure 3. The significance of pore density and solvents was compared by comparing *p*-values.

Total combinations = Number of solvent levels × Number of pore density levels = 3 (solvents) × 3 (pore density) = 9 combinations.

### 2.3. A Protocol for Statistical Calculations

To evaluate the effect of pore density and solvent type on the compressive strength and modulus, a two-way Analysis of Variance (ANOVA) was conducted in Microsoft Excel 2019 using the Data Analysis ToolPak add-in. With pore density and solvent type as the independent variables as well as compressive strength and the compressive modulus as the dependent variables, the data were arranged in Excel to meet the specifications for a two-way ANOVA analysis. Compressive strength and the compressive modulus were analyzed independently using Excel’s “Data—Data Analysis—ANOVA: Two-Factor with Replication” option. The statistical significance of the main effects (solvent type and pore density) and their interaction for each dependent variable were assessed using a significance threshold (alpha) of *p* < 0.05. Lastly, *p*-values were recorded and documented, and then the result was interpreted based on these statistical values.

## 3. Results and Discussion

According to ASTM D 1622-98 [45], the apparent carbon foam density ($\rho_{a}$) is obtained by the mass-to-volume ratio of each sample [46,47]. Furthermore, the relative density ($\rho_{r}$) is the ratio of carbon foam to the density of the solid material ($\rho_{s}$) from which the carbon foam was made. The cross-sectional area of the samples was calculated from the dimension of the samples (width and length) and its result is shown in Table 1. This cross-sectional area was used to determine the compressive stress from the stress–strain diagram of carbon foam.

### 3.1. Microstructural Analysis of Carbon Foam

**Figure 4:**
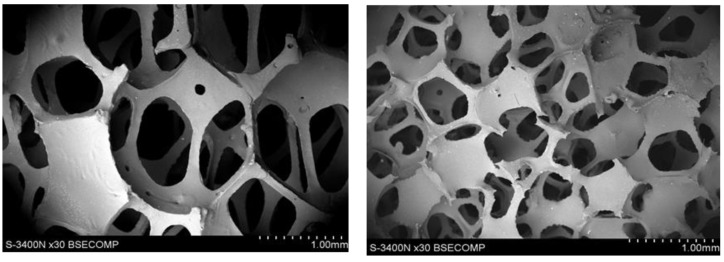
The structure of unmodified carbon foam, SEM.

**Figure 5:**
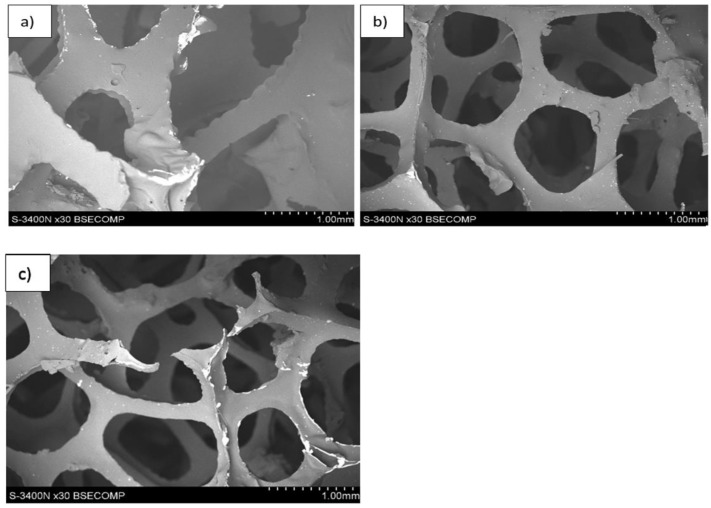
Structure of the 20 (**a**), 40 (**b**), and 60 (**c**) ppi foam samples studied, while modified FF resin with 20% ethanol was implemented, SEM.

**Figure 6:**
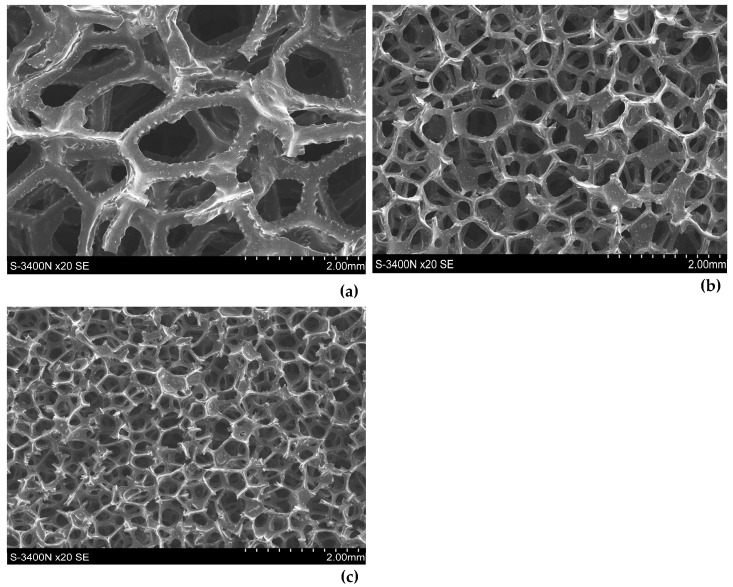
The structure of the 20 (**a**), 40 (**b**), and 60 (**c**) ppi foam specimens studied while modification with 40% methanol was implemented, SEM.

During heating and carbonization, the shape and pore size of polyurethane-derived carbon foam change, and melting could even occur. To prevent this, polyurethane foams were initially covered with a thin layer of a thermosetting polymer (in this case, a phenol–formaldehyde resin) to prevent changes in the shape and size of the foam cells. An important parameter to ensure the formation of only a thin coating that did not change the size of the pores (cells) was largely the viscosity of the polyurethane resin. Its high viscosity not only caused the formation of a thick layer on the cell walls but also contributed to closing the cell openings, which hindered the flow of the medium through the foam, which reduced flow and the possibility of infiltration. In addition, it is particularly important when such foam is used as reinforcement in the composite, which was not our case. The initial resin had a viscosity of ~1800 cP, which caused the openings to close in the foam cell (Figure 4). Therefore, an attempt was made to reduce its viscosity using diluents with low boiling points: methyl alcohol CH_3_OH, ethylene alcohol C_2_H_5_OH, and acetone CH_6_O. The initial resin was diluted with additions of 20%, 40%, or 20% ethanol, methanol, and acetone, respectively. When diluted with alcohols, the phenolic resin solutions obtained viscosities of 700 and 900 cP, respectively. Additionally, a 20% addition of acetone resulted in a viscosity of ~750 cP. At lower alcohol concentrations, some of the foam cell openings were closed, especially for the 60 ppi foam (Figure 5). However, after addition of 40% methanol or 20% acetone, the structure of the foams was correct, with no visible closed cell openings (Figure 6). Polyurethane foams infiltrated with the diluted resin were subjected to heat treatment to control the lattice process of the FF resin.

**Figure 7:**
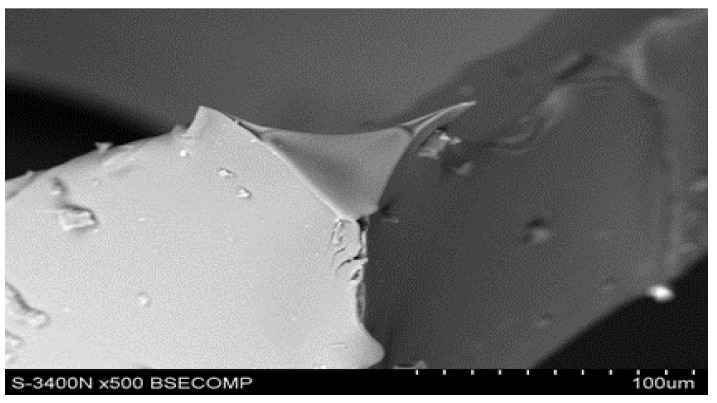
Fracture surface for a specimen after carbonization, SEM.

As shown in Figure 7, the fracture surface of the polyurethane-derived carbon foam was smooth with sharp, clean edges. This is indicative of a brittle fracture of polyurethane-derived carbon foam after carbonization. The sharpness of the edges in the image also indicates that the material likely failed under low-energy impact or stress, common in carbonized materials that have high brittleness. Additionally, the smooth and sharp fracture surface suggests that carbonization resulted in high structural integrity of the carbon foam but low toughness; that means it likely withstands compressive forces but may fail under tensile or impact stresses.

**Figure 8:**
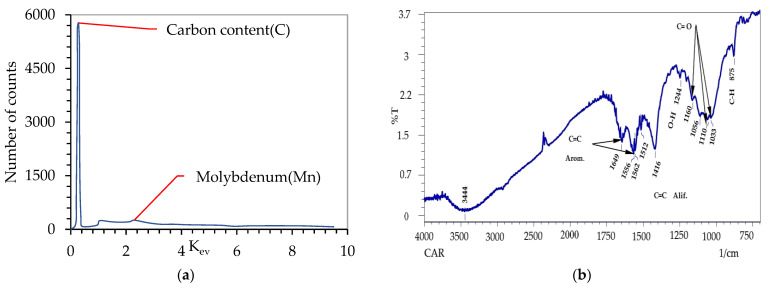
Chemical composition of the foam sample studied with EDS method (**a**) and IR analysis (**b**).

The foams with resin coating were heated at 69 °C for 4 h to prevent rapid evaporation of the diluent and the formation of blisters and pores of the coating. Subsequently, the foam precursors were cured at 160 °C for 6 h. The foam precursors were subjected to pyrolysis at 1000 °C in an argon atmosphere at a heating rate of 30–50 K/h and stable heating at the critical temperatures at which the most intense evaporation of the compounds included in the foam and coating took place. Carbonization left a carbonaceous material, shown in Figure 8. IR analysis shows that the foam contains strong C=C tensile bonds, characteristic of aromatic (1450–1610) and aliphatic (1600–1680) compounds, C–O bonds (formed because of the oxidation of glass carbon after pyrolysis), as well as deformation bonds of C–H aromatic compounds, and O–H groups characteristic of alcohols. This was confirmed by X-ray analyses via EDS. A homogeneous cross-section was obtained of the cell walls, with no clear differentiation between the foam and the coating material. An EDS analysis of the chemical composition of the foam after carbonization was performed. Those results indicated that 96% of the chemical composition of polyurethane-derived carbon foam was carbon and other elements were removed during the carbonization of carbon foam. Furthermore, only a small percentage of molybdenum was detected, a residue of the foam manufacturing process [5].

**Figure 9:**
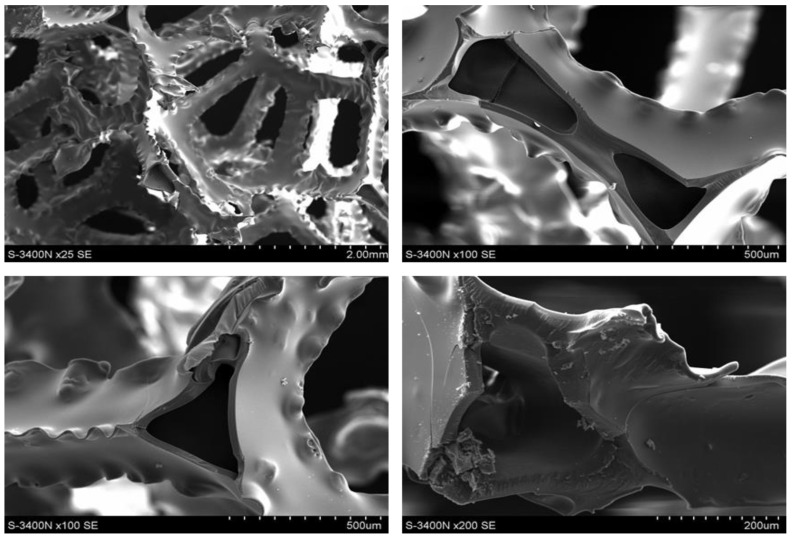
Structure and fracture surfaces of 20 ppi carbon foam, SEM.

**Figure 10:**
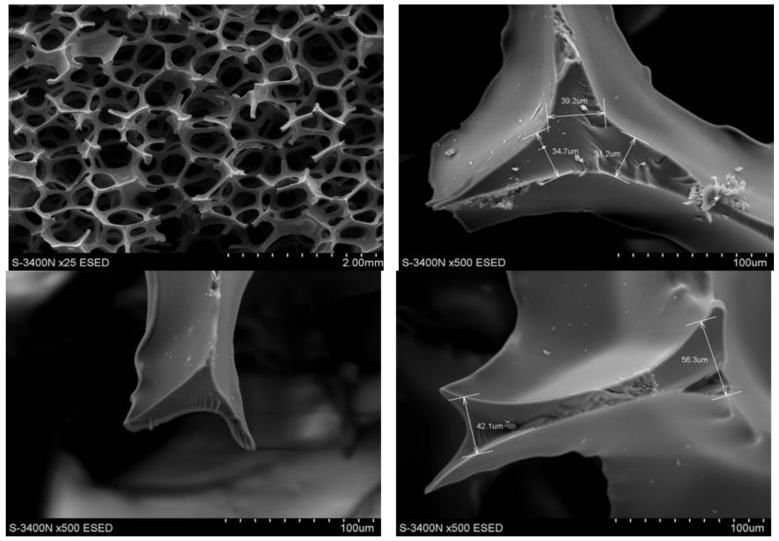
Structure fracture surfaces of 60 ppi carbon foam, SEM.

As shown in Figure 9, 20 ppi carbon foam has larger and more irregular pores with thicker struts. This results in a stronger but less intricate structure of carbon foam. Additionally, the fracture surface of 20 ppi was rough and irregular. Thicker struts and walls lend themselves to load-bearing applications. In contrast (Figure 10), 60 ppi carbon foam has smaller and more uniform pores with thinner struts, which result in denser walls and higher surface areas but lower mechanical strengths. Its fracture surfaces are smoother and more consistent, indicating greater brittleness. This foam is better suited for applications that require a high surface area, such as filtration or catalysis.

### 3.2. Compressive Strength

**Figure 11:**
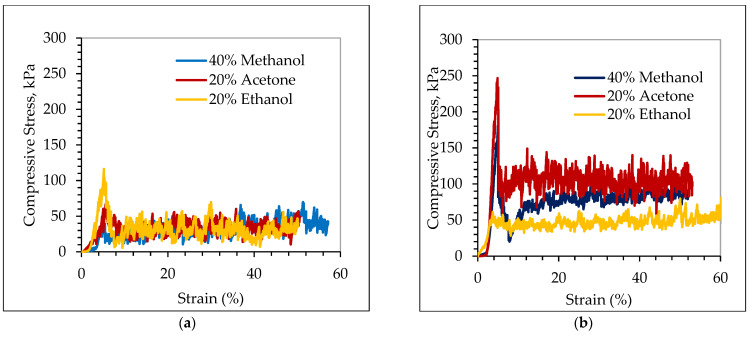

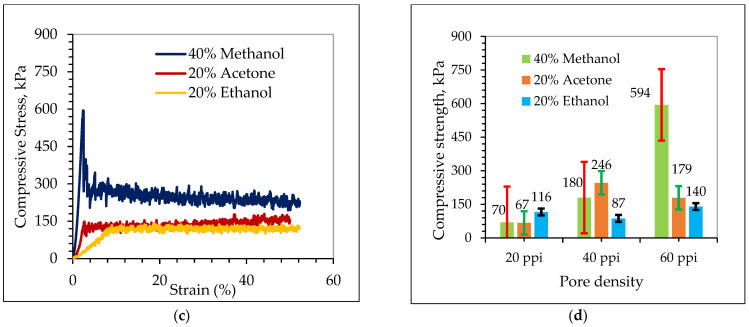
Compressive strength of 20 (**a**), 40 (**b**), and 60 (**c**) ppi and average ultimate compressive strength (**d**) with different solvents.

Compressive strength refers to the ability of a material to withstand compressive forces without breaking or deforming permanently and it is given by dividing the compressive force by the carbon foam cross-sectional area. The compressive strength responses of the carbon foams are shown in Figure 11.

As shown Figure 11a–c, the initial high slope corresponds to the elastic deformation, and the beginning of the nonlinearity on the stress–strain curve is the time when crack propagation in the pore walls happens [42]. These curves show a multistage deformation mechanism, including a linear elastic regime, a plateau region of roughly constant stress, and final failure [48]. The stress–strain curves started with a linear elastic phase where the strain was less than 5% [49,50].

The highest compressive stress was observed at the very beginning of the compression test, and is elastic in nature [13,51]. This is probably due to surface irregularities and slight misalignments, which create a high apparent stress as the testing machine presses the material [35]. Furthermore, in porous or cellular materials, initial compression can cause pore walls to align, compact, or re-orient slightly without fully collapsing the pores. This pre-compaction requires a high initial force because the pore walls are still relatively intact and resist deformation elastically. This can create a sharp initial peak in compressive stress, after which the stress might reduce as the structure begins to deform more uniformly [52]. Moreover, in some brittle materials (like carbon foams), microcracks are present before any compression load is applied. When compression starts, these microcracks need a high compressive force to be closed. This closing of microcracks results in peak stress because the material resists the load as it pushes these microcracks together. After the microcracks are fully closed, the stress drops slightly, and the material compresses more smoothly [53].

After stress reaches the yield values, stress starts to decrease. This decrement in stress is due to the following reason: the thin walls of the foam structure around the pores can no longer support the increasing load uniformly. This causes the walls to start collapsing or buckling, which reduces the material’s ability to withstand additional stress. On other hand, when the foam structure starts to break down, it dissipates energy through the re-arrangement and collapse of the cell walls. This re-arrangement of the cell walls temporarily reduces the stress in the material, which can cause the observed decrement in the stress–strain curve [54,55]. Moreover, the stress–strain diagram shows a nonlinearity (combination of peaks and valley) response. This nonlinearity is due to the random arrangement of cells and the variations in wall thickness, collapse and densification of its cellular structure, microcracking, and plastic deformation [47].

Figure 11a–c shows the stress–strain curves for 20, 40, and 60 ppi carbon foams subjected to quasi-static compressive loading with different pore densities and solvents. Additionally, as shown in Figure 11d, the maximum compressive strength was observed at a higher pore density (60 ppi) with 40% methanol solvent. This is because higher-pore-density cellular solids lead to a higher compressive strength, as presented in a paper [13]. In view of the material structure, this observation can be related to good interfacial bonding between the carbon foam and methanol at a higher pore density. This higher bonding can prevent a crack from propagating in the cell walls and ligaments of carbon foam [56]. Conversely, a lower compressive strength was observed at 20 ppi with 20% acetone solvent, which may result from lower interfacial bonding between acetone and the carbon foam skeleton at a lower carbon foam pore density [57].

### 3.3. Compressive Elastic Modulus (E)

**Figure 12:**
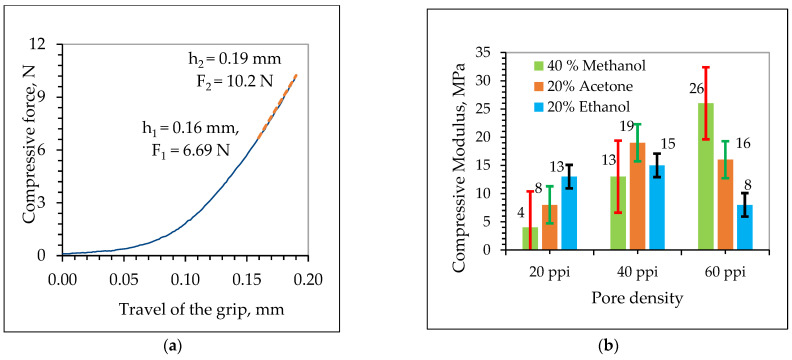
Elastic modulus representation (**a**) and average elastic compressive modulus of carbon foam across different solvents (**b**).

The elastic compressive modulus, often referred to as the compressive modulus or Young’s modulus in the compression of foam, measures its stiffness under compression and indicates how much the foam deforms under a given compressive load. The elastic compressive modulus is defined as the slope of the linear part of the curve as shown in Figure 12a [31]. The blue line represents compressive force versus travel of grip and red dot line is slope between two points used to determine elastic compressive modulus of carbon foam. 

As shown in Figure 12b, a higher compressive modulus was seen at a higher pore density (60 ppi) with 40% methanol solvent. This higher elastic modulus is due to the following reason: fine pore structures (60 ppi) allow better load distribution due to the increased surface area and connectivity of the cell walls, which results in greater resistance to deformation compared to the 20 and 40 ppi carbon foam [21,58]. In contrast, a lower pore density (20 ppi) with 40% methanol results in a lower elastic modulus due to its coarser pore structure, which reduces connectivity and load-bearing capability [59]. Additionally, in coarse foams with a lower density (20 ppi), solvents like methanol may not fill the larger pores effectively, resulting in less structural reinforcement and lower stiffness [60].

There are several models to predict the mechanical properties of foam. To determine elastic characteristics of these foams, the most applicable and widely used model by Gibson and Ashby was implemented. This model is based on the definition of a simple cubic unit cell and the use of beam theory to predict the elastic properties of cellular solids like foams. In the case of carbon foams with open cells, the variation in the elastic modulus with density is modelled by Gibson using Equation (1) [61].

(1) $$\frac{E_{cf}}{E_{s}}\approx {\left(\frac{\rho_{cf}}{\rho_{S}}\right)}^{2}$$

where $E_{cf}$ is the elastic modulus of the carbon foam, $E_{s}$ the elastic modulus of the solid material of which the foam is made, $\rho_{cf}$ is the density of the carbon foam, and $\rho_{S}$ is the density of the solid material. The carbon foam made in this study was made from polyurethane polymer foam. Equation (2) indicates that the elastic modulus of the foam depends only on the density of the foam given the solid material, and the relationship is known once a single parameter, $C_{2}$, is identified.

(2) $$\frac{E_{cf}}{E_{s}}=C_{2}{\left(\frac{\rho_{cf}}{\rho_{S}}\right)}^{2}$$

The value of C_2_ for 20, 40, and 60 ppi with 40% methanol solvent was 0.94, 1, and 1.03, respectively. Also, the value of C_2_ for 20, 40, and 60 ppi with 20% acetone was 0.91, 0.99, and 1.07, respectively. Furthermore, 1, 1, and 0.99 are the other values of C_2_ observed in 20, 40, and 60 ppi with 20% ethanol as solvent. All of these results agree with the statement reported by the Ashby and Gibson model, which states that the value of the proportionality constant ($C_{2}$) should be close to unity for open-cell foam [58].

### 3.4. Statistical Analysis of Properties of Carbon Foam

As shown in the data in Figure 11d and Figure 12b, and Table 1, higher variability of the elastic modulus (9), compressive strength (251.62), and relative density (0.007) of the carbon foam was observed in the 60 ppi carbon foam across different solvents. This indicated that the effects of solvents on the compressive strength, compressive modulus, and relative density were more significant in 60 ppi than 20 and 40 ppi. This means that solvents impacted the 60 ppi carbon foam’s compressive strength, compressive modulus, and relative density more than 20 and 40 ppi. This is due to large number of cell wall interactions with solvents, which results in a significant effect of solvents on compressive strength and the compressive modulus. In comparison, a lower variability in the elastic modulus (3.1) and relative density (0.003) was observed in the 40 ppi carbon foam. This means that the effect of solvents on the compressive modulus and relative density at 40 ppi is not as significant as compared with 20 and 60 ppi. Likewise, the lower variability in the compressive strength (27.47) was observed in the 20 ppi carbon foam. From these data, the effect of solvent on compressive strength was lower for 20 ppi than for either 40 ppi or 60 ppi. This is because in coarser foams (20 and 40 ppi), fewer cell walls interact with solvents, which results in significant effects of solvents on the compressive strength and compressive modulus of carbon foam.

Similarly, it is possible to compare which solvents (methanol, ethanol, or acetone) affect the compressive strength, compressive modulus, and relative density of carbon foam based on variable pore densities. Again, based on the data in Figure 11d and Figure 12b, and Table 1, a higher inconsistency of the elastic modulus (11.1), compressive strength (276.3), and relative density (0.01) of carbon foam were observed with 40% methanol solvents. This indicates that pore density significantly affects the compressive strength, compressive modulus, and relative density when using methanol as solvent. This is because methanol solvent has a smaller molecular size, higher polarity, stronger interaction with carbon, and better retention within the foam, which results in a significant effect on the compressive strength and modulus of carbon foams compared to other solvents. Conversely, 20% ethanol provides lower variability in the compressive strength (26.54), compressive modulus (3.6), and relative density (0.0036). This result indicates that the effect of pore density on the compressive strength, compressive modulus, as well relative density is not significant when 20% ethanol is used as solvent. Detail statistical analysis of above observation was shown in Table A1.

### 3.5. Two Factor ANOVA-Analysis

As shown in Table 2, the *p*-values for the elastic modulus with respect to pore density and solvents were much less than 0.05. This indicated that the effect of both solvents and pore density on the compressive modulus as well as compressive strength were significant. Additionally, the *p*-values for interaction (solvents and pore density) for both the elastic modulus and compressive strength were less than 0.05. From this result, it is easily observed that the effect of pore density and solvents on compressive strength as well as the compressive modulus were dependent on each other. Lastly, the *p*-values for the constant C_2_ were 0.187 and 0.976 across solvents and pore densities, respectively. This result indicates that neither pore density nor solvents affect the value of C_2_.

## 4. Conclusions

This article describes an experimental investigation on carbon foams of different foam pore densities and solvents under quasi-static compression loads. Both compressive strength and the compressive modulus are affected by pore density as well as the type of solvents. From the results, a higher compressive strength and elastic compressive modulus were observed at a higher pore density (60 ppi) with 40% methanol solvents. The higher compressive strength is because there is good interfacial bonding between the carbon foam and methanol at a higher pore density. This higher bonding prevents crack propagation in the cell walls and ligaments of the carbon foam. Conversely, a higher compressive modulus was due to the fine pore density allowing better load distribution due to the increased surface area and connectivity of the cell walls. On other hand, a lower compressive strength and compressive modulus were observed at a lower pore density. This is because at a lower pore density, there are few connections between cells and the load is distributed over a small area, which may cause less resistance to compressive load. Additionally, a higher stress concentration due to thicker cells may lead to uneven deformation under compressive loads and potentially earlier failure of the structures. Lastly, the carbon foam was mostly elastic, which fits well with the Ashby and Gibson model because the value of C_2_ was close to unity. While studying this kind of cellular solid, our carbon foam, several limitations were faced. Firstly, measurements of the outer dimensions produce errors due to the foam’s irregularity, porous structure, variations in pore size, and roughness of surface. This makes it difficult to understand the internal deformations and stress–strain responses clearly. Secondly, manufacturing itself causes the internal structure to differ in its dimensions and elasticity. Future research will focus more on improving microstructural analysis techniques by implementing micro-CT scanning or electron microscopy. Also, it is planned to explore additive manufacturing for customized porosity and geometry and study the effects of temperature on carbon foam durability.

## Figures and Tables

**Table 1:** Dimensions and properties of the selected studied samples.

	Solvents	Width, mm	Thickness, mm	Height, mm	Mass, g	Cross-Sectional Area, mm^2^	Volume, mm^3^	Apparent Density, kg/m^3^	Relative Density
20 ppi	40% Methanol	13	16	20	0.067	208	4160	16	0.014
	20% Acetone	16	18	20	0.132	288	5760	23	0.02
	20% Ethanol	12	12	16	0.065	144	2304	28	0.024
40 ppi	40% Methanol	12	17	20	0.114	204	4080	28	0.024
	20% Acetone	16	12	18	0.118	192	3456	34	0.030
	20% Ethanol	12	12	18	0.078	144	2592	30	0.026
60 ppi	40% Methanol	10	10	20	0.078	160	3200	39	0.034
	20% Acetone	11	17	20	0.026	187	3740	30	0.026
	20% Ethanol	17	20	20	0.019	340	6800	22	0.019

**Table 2:** Results of the two-factor ANOVA analysis.

Source of Variation	Compressive Strength	Compressive Modulus
	*p*-Values	
Pore density	2.04 × 10^−16^	1.14 × 10^−12^
Solvents	1.85 × 10^−14^	1.03 × 10^−4^
Interaction (pore density + solvents)	1.80 × 10^−16^	1.12 × 10^−13^

## Data Availability

The original contributions presented in the study are included in the article, further inquiries can be directed to the corresponding author.

## Appendix A

**Table A1:** Properties of carbon foams and a statistical analysis.

**Compressive Modulus, MPa**					
**Pore density (PPI)**	**Solvents**			**Statistic analysis**	
	40% Methanol	20% Acetone	20% Ethanol	Means (rows)	Standard deviation (rows)
20	4	8	13	8.3	4.5
40	13	19	15	15.67	3.1
60	26	16	8	16.67	9
Mean(columns)	14.33	14.33	12		
Standard deviation (columns)	11.1	5.7	3.6		
**Compressive Strength, kPa**					
**Pore density (PPI)**	**Solvents**			**Statistic analysis**	
	40% Methanol	20% Acetone	20% Ethanol	Mean (rows)	Standard deviation (rows)
20	70	67	116	84.33	27.47
40	180	246	87	171.00	79.88
60	594	179	140	304.33	251.62
Mean (columns)	281	164	114.33		
Standard deviation (columns)	276.3	90.44	26.54		
**Relative Density**					
**Pore density (PPI)**	**Solvents**			**Statistic analysis**	
	40% Methanol	20% Acetone	20% Ethanol	Mean (rows)	Standard deviation (rows)
20	0.014	0.020	0.0204	0.079	0.005
40	0.024	0.030	0.026	0.027	0.003
60	0.034	0.026	0.019	0.026	0.007
Mean (columns)	0.02	0.03	0.08		
Standard deviation (columns)	0.01	0.0048	0.0036		
**Constant C_2_**					
**Pore density (PPI)**	**Solvents**			**Statistic analysis**	
	40% Methanol	20% Acetone	20% Ethanol	Mean (rows)	Standard deviation (rows)
20	0.94	0.91	1.0	0.95	0.046
40	1.0	0.99	1.0	0.997	0.0058
60	1.03	1.07	0.99	1.03	0.04
Mean (columns)	0.99	0.99	0.997		
Standard deviation (columns)	0.0458	0.08	0.0058		
